# Contoured, prefabricated foot orthoses demonstrate comparable mechanical properties to contoured, customised foot orthoses: a plantar pressure study

**DOI:** 10.1186/1757-1146-2-20

**Published:** 2009-06-16

**Authors:** Anthony C Redmond, Karl B Landorf, Anne-Maree Keenan

**Affiliations:** 1 Section of Musculoskeletal Disease, University of Leeds, 2nd Floor, Chapel Allerton Hospital, Harehills Lane, Leeds LS7 4SA, UK; 2 NIHR Leeds Musculoskeletal Biomedical Research Unit, University of Leeds, 2nd Floor, Chapel Allerton Hospital, Harehills Lane, Leeds LS7 4SA, UK; 3 Department of Podiatry, Faculty of Health Sciences, La Trobe University, Bundoora, 3086, Australia; 4 Musculoskeletal Research Centre, Faculty of Health Sciences, La Trobe University, Bundoora, 3086, Australia

## Abstract

**Background:**

Foot orthoses have been demonstrated to be effective in the management of a range of conditions, but there is debate as to the benefits of customised foot orthoses over less expensive, prefabricated devices.

**Methods:**

In a randomised, cross-over trial, 15 flat-footed participants aged between 18 and 45 years were provided with semi-rigid, customised orthoses and semi-rigid, contoured, prefabricated orthoses. Pressures and forces were measured using an in-shoe system with subjects wearing shoes alone, wearing customised orthoses, and again when wearing contoured prefabricated orthoses. Two weeks acclimatisation was included between cross-over of therapy. Repeated measures ANOVA models with post-hoc, pair-wise comparisons were used to test for differences.

**Results:**

When compared to wearing shoes alone, wearing either the customised orthoses or the prefabricated orthoses was associated with increases in force and force time integrals in the midfoot region. Peak and maximum mean pressure and pressure-time, and force-time integrals were reduced in both the medial and lateral forefoot. There were, however, no significant differences between the customised orthoses and the prefabricated orthoses at any site.

**Conclusion:**

There was a similar change in loading with both the semi-rigid customised and the semi-rigid prefabricated orthoses when compared to the shoe alone condition. However, while customised devices offered minor differences over prefabricated orthoses in some variables, these were not statistically significant. The results suggest that there may be only minor differences in the effects on plantar pressures between the customised and the less expensive prefabricated orthoses tested in this study, however further research is warranted.

## Background

Functional foot orthoses are reportedly helpful to patients with a range of lower limb musculoskeletal problems [1-5] and are widely prescribed [6]. It is generally thought that the use of foot orthoses is associated with systematic alterations in the mechanics of the feet and lower limb [7-9], and it has been established that orthoses alter plantar pressures systematically [10-13]. The issue of the cost effectiveness of providing foot orthoses has been raised [14], as the cost of foot orthoses represents a considerable burden to patients, clinicians and health providers alike.

In clinical practice, a range of customised, and less expensive prefabricated orthoses are prescribed [6]. Customised foot orthoses are semi-rigid devices, made to a cast of the patient's foot with an individualised prescription, and are generally considered the gold standard [6]. Prefabricated orthoses are mass produced devices made to fit to a generic foot shape, and include a variety of flat insoles, arch supports, and single plane wedges; as well as contoured devices that mimic many of the physical characteristics of customised devices. Results from recent clinical trials have demonstrated that some contoured prefabricated devices are as beneficial in reducing patient symptoms as more expensive customised orthoses for certain conditions [5,15,16]. The relative mechanical influences of customised and prefabricated orthoses are less clear however. To date, most research studies have focussed on "gold standard" customised orthoses, and little data has been published on the mechanical effects of prefabricated devices.

Customised semi-rigid orthoses have been shown to cause different mechanical effects than the cheapest prefabricated alternative, single-plane wedges. It is not appropriate therefore, to consider single-plane wedging comparable to provision of customised orthoses in clinical practice [13]. Single-plane wedges differ significantly from custom devices in their physical characteristics, however, as well as in the process of dispensing them off-the-shelf. Contoured prefabricated devices offer an intermediate approach, as they have physical characteristics closer to those of customised orthoses, but are provided without the costs of the customisation process.

The aim of this exploratory study was to evaluate differences in the magnitude and timing of plantar pressures and forces occurring with the use of semi-rigid, cast functional foot orthoses and semi-rigid, contoured, prefabricated orthoses.

## Methods

### Participants

The study was conducted at the University of Western Sydney between October 2002 and July 2003, with ethical approval granted by the University of Western Sydney Human Ethics Committee. Fifteen participants, aged 18–45 and with a flat foot type were recruited through the polyclinic via a poster campaign. The sample size in the study provides greater than 80% power to detect a difference between the orthoses in the force time integral of 29 N.s, incorporating a standard deviation of 27 N.s and an alpha level of 0.05 (force time integral data taken from a previous study using a similar protocol [13]).

All participants met the inclusion criteria of a relaxed calcaneal stance position of > 5° valgus, plus a Foot Posture Index score of greater than eight from a maximum score of 16 [17] and a score on Rose's Valgus Index of >18 [18,19]. The validity and reliability of the Foot Posture Index and Rose's Valgus Index have been described [17,18,20-22] and a range of measures was used to ensure rigorous screening for appropriate foot postures. To ensure that gait and plantar pressures were not influenced by current pain or disability, participants were otherwise healthy. Patients with a history of overuse or traumatic injury to the lower limb in the past 6 months, a history of bony surgery to the lower limb, or with a systemic endocrine, neurogenic or musculoskeletal disorder were excluded.

### Orthosis type

To allocate orthosis type, sealed-envelope randomisation was employed, with intervention cross over. Following enrolment into the study, the participants underwent a standardised prone casting protocol to obtain neutral impression casts [23]. The customised orthosis was a 'modified Root' type orthosis, posted to the neutral calcaneal stance position. This device was chosen as it was the most common prescription used in Australia and New Zealand at the time of the study [6]. The customised orthoses were manufactured at a commercial orthosis laboratory (The Orthotic Laboratory Pty Ltd, Melbourne, Australia) according to a strictly defined procedure and under the care of a single, experienced technician. The shell material was 4 mm white 'semiflex' polypropylene, heel posts were made from 450 kgm^3 ^ethyl vinyl acetate (EVA), machined as appropriate to the clinician's prescription, and a thin vinyl top cover was added.

The prefabricated devices were a contoured device made to a standardised last rather than a custom plaster mould. The prefabricated devices were a commercially available brand (Cast and Foot Adjusted Orthoses^®^) supplied by the same laboratory (The Orthotic Laboratory Pty Ltd, Melbourne, Australia) and each device incorporated a 4° varus rearfoot post. Materials used for the manufacture of the prefabricated orthoses were the same as used for the customised devices. Comparisons of the two devices are presented in additional file 1.

### Data capture

Plantar pressures and forces were obtained for the right foot of each participant, using the Pedar in-shoe pressure system (Novel GmbH, Munich, Germany). This system has been described in the literature [24-26] and has been used in the evaluation of orthotic function previously. [11,13]. In order to avoid problems associated with dependency-related effects that can arise when using two limbs from the same person [27], data from the right limb alone were recorded.

Participants were assessed using a standardised protocol. Gait speed and cadence were recorded using a stopwatch and metronome while participants walked for three minutes until a comfortable gait speed and cadence were established. Subsequent analyses used this standardised cadence and gait speed for all measures to ensure parity between conditions. All measures were made using the Pedar insoles of appropriate size fitted to a pair of lightweight canvas Dunlop Volley sneakers (Dunlop Australia Pty Ltd, Melbourne, Australia), from which the inner sole/linings had been stripped to create a lightweight shoe, assumed to have only a minimal effect on foot function. Measures were undertaken with participants wearing either the standard shoe only or the standard shoe and the appropriate test device.

Baseline pressure and force measures were obtained prior to issue of the trial devices to avoid cumulative adaptive affects that might have occurred once orthosis wearing commenced. Participants were then randomised according to a computer generated randomisation protocol, to wear one type of orthosis for at least two-weeks prior to returning for plantar pressure and force measures. Participants then crossed over to the alternative orthosis and repeated the two-week run-in period before returning for further measurement.

Participants walked for three laps of a nine meter walkway at a controlled gait speed and cadence [28]. Pedar data were sampled at 50 Hz. Turning steps, and acceleration steps were identified from a pause included in the clinical protocol and the characteristics of resulting force-time curves. They were deleted using the Pedar step analysis software Novel win 0.87, so as to include only mid-lap steps in the analysis (Novel GmbH Munich, Germany) as described previously [13]. Valid steps (stance phase only) were derived at this point by applying a minimum threshold to the force time curves for each step in the step analysis software. Between 12 and 16 mid-lap steps were obtained per participant in each of the orthosis conditions.

### Analysis

**Figure 1:**
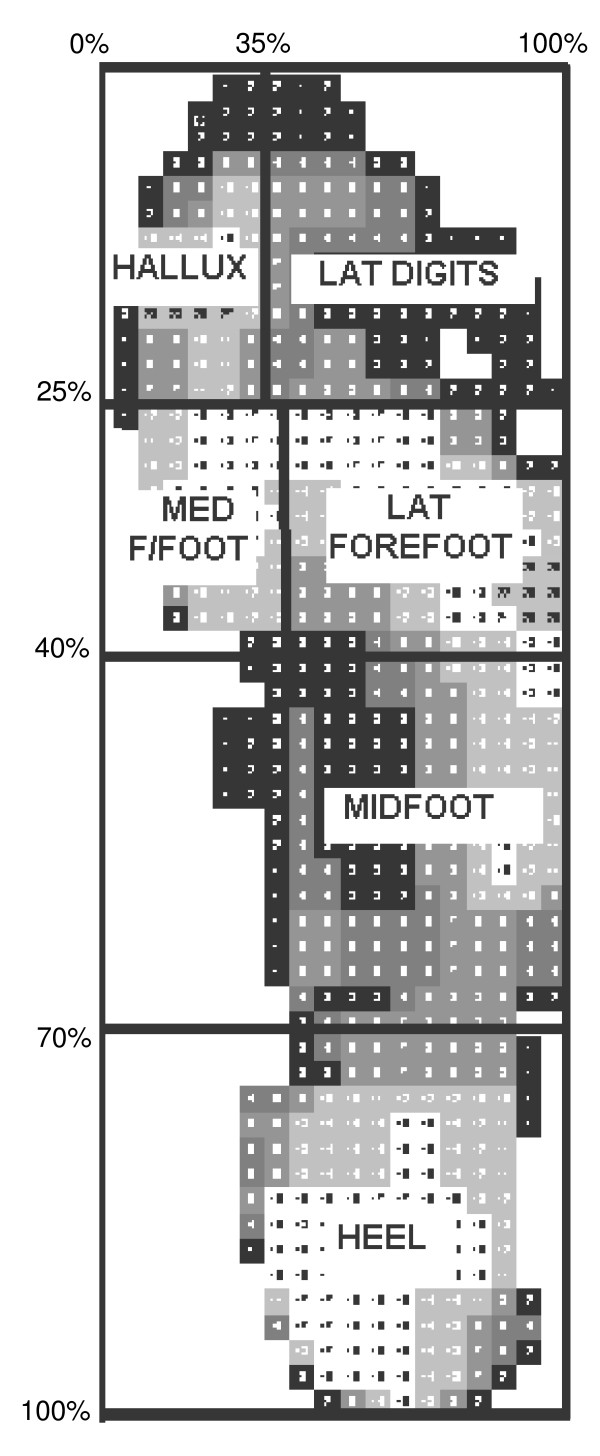
**The five mask areas defined by the percentage mask**. Note: the Lateral digits mask was defined but not included in the analysis.

Data were compared for five mask regions (Figure 1) corresponding to anatomically relevant areas of the foot, namely the heel, midfoot, medial forefoot (first metatarsophalangeal joint), lateral forefoot (2–5^th ^metatarsophalangeal joints), and hallux. Data from the lateral digits yielded low values with high variance. Because of the potential for error and limited importance of the lateral digit mask area, this mask was excluded from the subsequent analysis. Variables of interest were extracted from each of the five mask areas. It is not yet known precisely which measures of force and pressure are most meaningful in the clinical setting so, for completeness, a broad range was described in full. These included pressure (maximum mean pressure, peak pressure), force (maximum force, mean force), spatial (contact area) and temporal (duration of loading as a proportion of total foot contact) variables. The integrals of force and pressure were also investigated.

Comparisons were made between the group mean values of the three conditions: (i) the patient in the shoe only (control) condition; (ii) while wearing the prefabricated orthoses and; (iii) while wearing the customised orthoses. Preliminary plotting and tests were undertaken to explore suitability of the data for parametric analysis. PP plots were examined and the data were interpreted against Mauchly's Test for Sphericity and found to be suitable. Repeated measures ANOVA models were used to determine the significance of the within-subject effects in each of the models, and post-hoc pair-wise comparisons were made using the Bonferroni adjustment for multiple comparisons.

## Results

Force and pressure measurements for each of the mask areas are presented in detail in Tables 1, 2, 3, 4 and 5, with a summary for the total foot area given in Table 6. Contact times were similar in the three test conditions indicating consistency of control in gait velocity during acquisition as shown in Table 7. Seven variables over five masked areas were analysed, resulting in 35 variable/mask combinations. The prefabricated orthoses and/or customised devices produced statistically significant and comparable mechanical changes relative to the control condition for 11 of the 35 variable/mask combinations. The customised orthoses showed enhanced changes over the prefabricated devices in three variables. However, the results for the customised and prefabricated devices did not differ statistically for any of the variable/mask region combinations.

**Table 1:** Mean (SD) values for the heel mask area (N = 15)

	**Custom FO [CFO]**	**Prefabricated orthoses (Prefab)**	**Control-Shoe only (SO)**	**Overall significance of model**
**Peak Pressure (kPa)**	219.7 (52.1)	251.7 (101.1)	265.3 (52.4)	F = 2.44, P = 0.105*
**Maximum Mean Pressure (kPa)**	126.2 (20.4)	135.0 (36.1)	139.6 (22.7)	F = 1.145, P = 0.318
**Pressure Time Integral (kPa.s)**	49.0 (14.3)	60.4 (25.1)	56.1 (14.2)	F = 1.929, P = 0.164*
**Maximum Force (N)**	506.4 (90.0)	509.2 (98.6)	528.1 (100.3)	F = 3.02, P = 0.065
**Force Time Integral (N.s)**	107.3 (34.0)	109.1 (34.0)	107.1 (32.2)	F = 0.315, P = 0.732
**Area (cm^2^)**	42.6 (2.1)	42.0 (3.0)	41.1 (2.1)	F = 3.209, P = 0.083
**Time (% rollover)**	73.3 (12.1)	78.0 (11.3)	70.1 (13.3)	F = 4.181, P = 0.042

*Adjusted significance of difference between SO and CFO condition P < 0.05

There were no significant pair-wise differences between the Prefab and CFO condition or between the Prefab and SO condition

**Table 2:** Mean (SD) values for the midfoot mask area (N = 15)

	**Custom-FO (CFO)**	**Prefabricated orthoses (Prefab)**	**Shoe only (SO)**	**Overall significance of model**
**Peak Pressure (kPa)**	165.8 (56.6)	171.5 (60.2)	183.0 (54.5)	F = 1.127, P = 0.313
**Maximum Mean Pressure (kPa)**	67.6 (16.6)	69.5 (14.4)	78.2 (15.7)	F = 7.929, P = 0.002^†^*
**Pressure Time Integral (kPa.s)**	56.1 (17.8)	57.4 (17.6)	58.9 (22.0)	F = 0.538, P = 0.521
**Maximum Force (N)**	264.7 (82.9)	253.6 (79.8)	206.0 (70.9)	F = 14.35, P < 0.001^†^*
**Force Time Integral (N.s)**	76.8 (34.6)	72.2 (29.5)	57.3 (27.6)	F = 16.44, P < 0.001^†^*
**Area (cm^2^)**	46.8 (8.7)	43.1 (9.3)	32.5 (8.2)	F = 51.387, P < 0.001^†^*
**Time (% rollover)**	96.2 (2.5)	96.0 (3.0)	92.6 (4.3)	F = 12.823, P = 0.001^†^*

^†^Adjusted significance of difference between SO and Prefab condition P < 0.05

*Adjusted significance of difference between SO and CFO condition P < 0.05

There were no significant differences between the Prefab and CFO condition

**Table 3:** Mean (SD) values for the medial forefoot (1^st ^MTP joint) mask area (N = 15)

	**Custom-FO (CFO)**	**Prefabricated orthoses (Prefab)**	**Shoe only (SO)**	**Overall significance of model**
**Peak Pressure (kPa)**	256.4 (74.1)	248.8 (68.4)	274.0 (86.8)	F = 3.775, P = 0.060
**Maximum Mean Pressure (kPa)**	128.8 (40.1)	126.1 (42.7)	136.7 (43.3)	F = 4.080, P = 0.028^†^*
**Pressure Time Integral (kPa.s)**	54.1 (17.1)	53.4 (18.9)	66.3 (17.4)	F = 24.369, P < 0.001^†^*
**Maximum Force (N)**	151.1 (63.1)	145.2 (65.8)	159.1 (66.3)	F = 2.861, P = 0.074
**Force Time Integral (N.s)**	29.5 (11.2)	28.1 (12.9)	36.9 (13.5)	F = 16.987, P < 0.001^†^*
**Area (cm^2^)**	12.3 (1.0)	12.1 (1.6)	12.2 (1.3)	F = 0.339, P < 0.715
**Time (% rollover)**	75.3 (10.5)	75.1 (12.1)	83.9 (8.1)	F = 14.662, P < 0.001^†^*

^†^Adjusted significance of difference between SO and Prefab condition P < 0.05

*Adjusted significance of difference between SO and CFO condition P < 0.05

There were no significant differences between the Prefab and CFO condition

**Table 4:** Mean (SD) values for the lateral forefoot mask area (N = 15)

	**Custom-FO (CFO)**	**Prefabricated orthoses (Prefab)**	**Shoe only (SO)**	**Overall significance of model**
**Peak Pressure (kPa)**	289.9 (78.1)	286.8 (78.6)	280.9 (63.1)	F = 0.482, P = 0.544
**Maximum Mean Pressure (kPa)**	139.5 (40.2)	143.3 (37.9)	146.0 (34.9)	F = 1.600, P = 0.220
**Pressure Time Integral (kPa.s)**	66.9 (21.0)	67.8 (24.9)	74.4 (21.7)	F = 8.639, P = 0.001^†^*
**Maximum Force (N)**	390.5 (116.6)	388.1 (117.6)	383.6 (109.8)	F = 0.309, P = 0.643
**Force Time Integral (N.s)**	87.5 (27.5)	88.3 (34.3)	97.2 (33.6)	F = 6.073, P = 0.006^†^*
**Area (cm^2^)**	29.1 (1.0)	28.7 (0.9)	27.9 (1.6)	F = 8.101, P = 0.002*
**Time (% rollover)**	89.4 (8.2)	89.3 (8.8)	92.1 (5.6)	F = 4.255, P = 0.024

^†^Adjusted significance of difference between SO and Prefab condition p < 0.05

*Adjusted significance of difference between SO and CFO condition p < 0.05

There were no significant differences between the Prefab and CFO condition

**Table 5:** Mean (SD) values for the hallux mask area (N = 15, DF = 2)

	**Custom-FO (CFO)**	**Prefabricated orthoses (Prefab)**	**Shoe only (SO)**	**Overall significance of model**
**Peak Pressure (kPa)**	193.4 (82.5)	194.9 (73.1)	206.2 (101.4)	F = 0.968, P = 0.392
**Maximum Mean Pressure (kPa)**	68.7 (27.8)	68.5 (31.3)	69.1 (26.1)	F = 0.020, P = 0.980
**Pressure Time Integral (kPa.s)**	40.2 (22.0)	40.6 (22.9)	47.7 (29.6)	F = 4.034, P = 0.046
**Maximum Force (N)**	95.4 (61.5)	95.3 (63.3)	93.0 (55.5)	F = 0.08, P = 0.864
**Force Time Integral (N.s)**	18.4 (16.1)	18.5 (16.3)	18.7 (15.3)	F = 0.031, P = 0.910
**Area (cm^2^)**	14.4 (2.6)	14.5 (2.4)	14.0 (3.0)	F = 0.781, P = 0.468
**Time (% rollover)**	68.8 (14.3)	69.2 (14.0)	73.2 (16.4)	F = 2.239, P = 0.125

There were no significant pairwise differences between any of the three conditions.

**Table 6:** Mean (SD) values for the total foot area (N = 15, DF = 2)

	**Custom-FO (CFO)**	**Prefabricated orthoses (Prefab)**	**Shoe only (SO)**
**Peak Pressure (kPa)**	335.9 (65.7)	350.8 (77.5)	346.9 (67.5)
**Maximum Mean Pressure (kPa)**	107.6 (15.8)	109.1 (17.8)	121.0 (18.2)
**Pressure Time Integral (kPa.s)**	108.9 (25.2)	116.3 (27.7)	122.3 (24.0)
**Maximum Force (N)**	846.7 (193.3)	840.9 (186.7)	803.6 (179.0)
**Force Time Integral (N.s)**	341.7 (89.8)	337.8 (90.2)	337.0 (89.0)
**Area (cm^2^)**	83.3 (11.5)	81.0 (11.0)	73.3 (10.4)

**Table 7:** Mean (SD) values for total contact time (N = 15, DF = 2)

	**Custom-FO (CFO)**	**Prefabricated orthoses (Prefab)**	**Shoe only (SO)**	**Overall significance of model**
**Total contact time in milliseconds**	569 (53)	564 (54)	567 (51)	F = 0.160, P = 0.853

**Figure 2:**
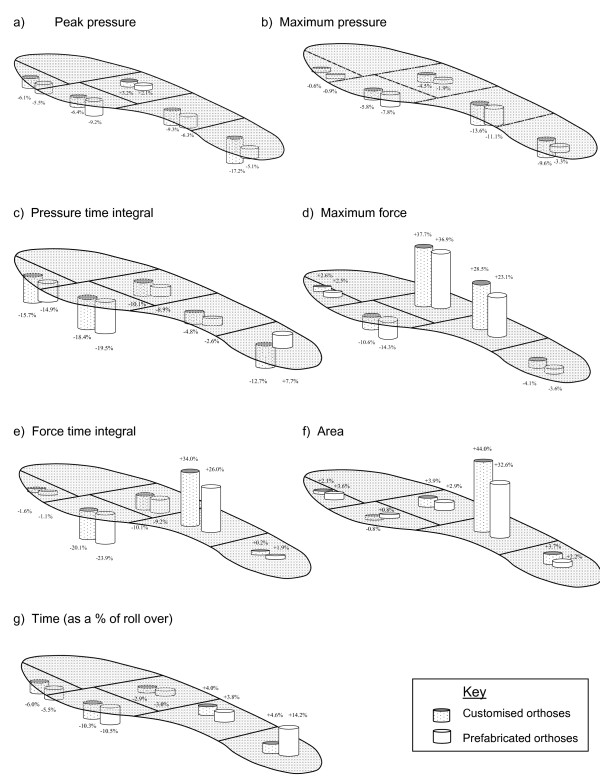
**Differences from control (shoe only) condition associated with wearing customised and prefabricated orthoses**.

Although the differences were not significant, the customised orthoses compared to the prefabricated devices produced decreased loading at the heel by up to 12% and increased the contact area of the midfoot (44% greater contact area than control for the customised orthoses, compared with 33% for the prefabricated devices) – Figure 2. The loading characteristics of the foot in response to both types of device, however, were comparable both at the midfoot (0.2% to 8% difference) and forefoot (0.2% to 3.7% difference).

### Heel mask area

There were no significant differences between the customised and the prefabricated insoles in any of the variables measured at the heel (Table 2). The customised devices reduced the mean pressure (9.6%) and peak pressure (17%) at the heel, which was more than for the prefabricated devices (3.3% and 5.1%), although the difference between the two orthoses was not statistically significant. Some of the difference observed between the two devices may be attributable to the increase in the duration of heel loading with the prefabricated devices, which contributes to a small increase in the pressure time integral and force time integral in the heel.

Neither device had a profound effect on the heel contact area or force, with change in these variables limited to 4%. The effect of the customised and prefabricated devices did not differ significantly for any variable.

### Midfoot

Both types of device alter midfoot loading considerably, with maximum force increased by 23% (prefabricated orthoses) to 29% (customised orthoses) over the control condition (Table 3). Both devices also contributed to a similar increase in the proportion of the gait cycle for which the midfoot region was loaded. Midfoot contact area was increased markedly by the use of either device (customised orthoses = 44% and prefabricated orthoses = 33%). This increase in midfoot contact area contributes to the pressure variables being reduced relative to the control condition, despite an increase in force through the midfoot.

### Medial forefoot

Both types of device produced moderate changes in the loading of the medial forefoot compared to control. Pressure and force variables fell 6% to 23%, and both devices also reduced the loading time for this region by 10% (Table 4). Consequently, force and pressure integrals were reduced by approximately 20% for both the prefabricated and the customised devices.

### Lateral forefoot

The maximum force transmitted through the lateral forefoot was reduced by some 36% to 37% by both prefabricated and customised devices (Table 5). The lateral forefoot demonstrated only minimal reduction in the duration of loading, and consequently the change in the lateral forefoot integrals for both types of orthoses versus control was 1.9% to 10.1%. This finding contrasts with the significant reduction in the integrals seen in the medial forefoot mask area.

The mechanical effects of the prefabricated and customised devices were similar at both the medial and lateral forefoot, with no variable differing by more than 4% between the two devices.

### Hallux

In agreement with previous data [13], a small increase in peak pressure (6%), duration of loading (6%) and pressure time integral (15% to 16%) can be seen under the hallux following the addition of either type of device (Table 6). Again, the mechanical effect of the two types of device was indistinguishable for variables in this region, with the differences between prefabricated and customised devices no greater than 1.5%.

## Discussion

This study aimed to compare the mechanical effects at the foot-orthosis interface, of two commonly used approaches to providing foot orthotic therapy. There is a growing body of evidence supporting the use of foot orthoses, typically customised foot orthoses, to prevent and manage a range of musculoskeletal complaints in the lower limb [2,4,5,29]. If similar effects may be achieved with less costly interventions there is potential for savings to the health system without compromise to the quality of care. This study concentrated on a comparison of the two approaches based on a series of objective mechanical variables, rather than on subjective patient-related factors such as pain or health-related quality of life [14]. The two device types were intentionally similar to ensure that any differences were due to the prescription and manufacturing process rather than material properties. There remains a need for further studies that compare devices made from different materials or to differing prescriptions.

Mean force, peak plantar pressures and pressure time integrals were consistent with that described in the literature previously [10,11,13]. Also in agreement with previous data [13], we found that the introduction of a contoured orthosis to the footwear resulted in a shift of load from the forefoot and rearfoot toward the midfoot, compared to the control condition. This effect was similar for both the prefabricated and customised devices tested, and contrasts with the absence of this effect in the single plane, prefabricated wedges evaluated previously [13]. The shift in load toward the midfoot is, in the case of contoured devices, associated with a concomitant increase in midfoot contact area, which minimises change in pressures in this region. The timing of the foot loading is altered, reinforcing the contention of Reed and Bennett [30] that part of the mechanical effect of a contoured orthosis arises through its action as a fulcrum at the midfoot, prolonging loading in this area.

The contoured prefabricated and customised devices clearly offered similar mechanical properties over most of the foot. None of the pressure force, area or timing variables differed by more than 12%, and the two types of device must be considered highly comparable from a mechanical perspective. At the heel, forces reduced by a similar amount from the control condition in both types of device, although small differences in heel pressures were found between the two devices. While there were no statistically significant differences detected between the orthosis types, the percentage changes from control suggest that the individualisation of the customised orthosis may be marginally beneficial in reducing heel pressures. This could have implications where offloading of the heel is the primary clinical aim of an orthosis prescription, however further research is warranted that more specifically focuses on this hypothesis.

The customised orthosis provided a greater increase in midfoot loading area (44% increase), although as the prefabricated devices also demonstrated a 33% increase in midfoot contact area, the additional benefits of customisation may be limited. In the forefoot, both types of device produced similar systematic changes compared with the control, suggesting that for forefoot complaints, the mechanical effects of prefabricated and customised devices might be comparable.

We note that the prefabricated orthosis used in our study was considerably less expensive than the customised device. Formal recommendations on cost effectiveness can only be made, however, on data from quality health-economic studies, in which the burden of disease and any alleviation associated with the interventions are evaluated in detail. Nevertheless, as the preparation and manufacturing are different between the two devices, it is appropriate to explore the issue of costs associated with both orthoses. The diagnostic assessment and follow-up protocols are similar for both the customised and the prefabricated orthoses. However, there are some differences in the costs incurred during the pre-manufacture stage. Customised devices require appropriate measurements (e.g. neutral calcaneal stance position) and extra time, materials and expertise to take the required neutral plaster cast. These costs are not incurred with prefabricated orthoses. Further costs are incurred at the manufacturing laboratory by the production of a positive cast and this is reflected in the cost to the practitioner. In this study the purchase price of the customised devices ($AUS 89.95) was two and a quarter times that of the prefabricated devices ($AUS 39.95). The labour, materials and laboratory costs incurred in this study indicate that at the point of issue, customised devices were 3.5 times more costly, and for the entire episode of care, 2.5 times more costly than the prefabricated devices.

In common with similar studies, there are protocol issues that also warrant further discussion. Participants in the study wore standard shoes during data capture but were free to wear their own footwear in the intervening periods. Footwear is known to influence lower limb function variably [31] and the current data do not necessarily apply to the broad range of footwear in use. Adaptive effects occurring during the acclimatisation period may not have been detected following transfer to the standard shoe and the use of standard shoes could be considered to provide undue homogeneity in the results.

Also warranting consideration is the choice of the 14 day acclimatisation period. It is known that the process of acclimatising to wearing orthoses in the early stages includes both mechanical and more complex neurophysiological adaptations [32]. Same-day or short-period pre and post intervention measures allow inadequate time for such adaptive changes to occur and we introduced a period intended to be both practical while being long enough to allow for adaptive changes. We note, however, that review periods in clinical practice can range from as little as one week to as long as many months and we recommend that future studies supplement the initial measures with longer term follow-up to further investigate adaptive response over time.

Two statistical issues warrant discussion when considering the results of this study. On the one hand, this was an exploratory study which aimed to describe the differences in loading that occur over the plantar surface of the foot in response to orthotic therapy. The results of inferential tests (ANOVAs) have been reported to indicate which variables demonstrated differences that are more likely to be of statistical significance. However, because it was an exploratory study, in which we did not pre-specify a primary hypothesis or hypotheses, many inferential (significance) tests were performed. There are important drawbacks in this approach, the most important being the increased possibility of Type I statistical errors (where insignificant effects are deemed significant because of cumulative probabilities associated with the conduct of multiple hypothesis tests). In an exploratory study such as this, the statistical significance of differences should be interpreted by the reader only as an indicator of those differences that are most likely to be of relevance. Such exploratory studies are hypothesis generating and usually lead to further study if significant findings emerge. If significant findings are found, the appropriate course of action is to then design a further study that focuses on those variables, thus reducing the number of hypothesis tests and chance of Type 1 error. With this in mind, further research would be beneficial that specifically targeted the most relevant of the variables explored in this study.

On the other hand, even though we attempted to recruit sufficient participants into the study – via an a priori sample size calculation – to have appropriate statistical power to detect important differences, the sample size at 15 was still relatively small. Accordingly, for some variables our data may be at risk of Type 2 statistical error where we concluded there were no statistically significant differences, even though there may have been clinically important differences but the sample size was insufficient to detect them. With the above two statistical issues in mind it would be (i) worthwhile confirming our significant findings with further studies, and (ii) ensure these studies have sufficiently large sample sizes using appropriate a priori sample size calculations.

Finally, in this exploratory study, we have evaluated the mechanical effects of two different types of devices, similar in material, with the main difference between the two the customisation process. Objective results from a study of the mechanical effects are important, but further studies incorporating a range of the broader, patient-reported factors such as symptom relief and comfort are also needed to further inform the debate.

## Conclusion

In this study both customised and prefabricated orthoses altered plantar loading in a shod foot compared to wearing a shoe without an orthosis. The customised device demonstrated minor differences over the prefabricated orthosis in some variables, but in no case were the differences statistically significant. This is in contrast to the significant differences between customised orthoses and single plane wedges evaluated in earlier studies, suggesting that the contouring of the arch of an orthotic device is influential, whether it is derived from a custom cast, or from the generic last used to form a prefabricated device.

While these data indicate that customised and prefabricated orthoses alter the plantar loading profile during walking, further research is required to ascertain whether one device affords a greater mechanical effect than the other. While previous work has suggested that single plane prefabricated orthoses cannot be considered a mechanical surrogate for custom orthoses, contoured prefabricated devices may address some of the shortcomings of single-plane devices without incurring the attendant costs of customisation.

## Competing interests

KBL is a Deputy Editor of Journal of Foot and Ankle Research. It is journal policy that editors are removed from the peer review and editorial decision-making processes for papers they have co-authored.

## Authors' contributions

ACR designed the study, secured funding for the study, supervised data collection, performed the data analysis and wrote the manuscript. KBL assisted with the design of the study, securing funding and writing of the manuscript. AMK assisted with the design of the study, securing funding and writing of the manuscript. All authors have read and approved the final manuscript.

## Supplementary Material

Additional file 1 **Characteristics of the customised and prefabricated orthoses**. The data compare the prescription and physical characteristics of the two types of device. Click here for file
