# Supplementary material for: Contoured, prefabricated foot orthoses demonstrate comparable mechanical properties to contoured, customised foot orthoses: a plantar pressure study
Source: J Foot Ankle Res. 2009 Jun 16;2:20. doi: 10.1186/1757-1146-2-20 (PMC2711934; doi:10.1186/1757-1146-2-20)
Supplement: Additional file 1 — Characteristics of the customised and prefabricated orthoses. The data compare the prescription and physical characteristics of the two types of device. [file 1757-1146-2-20-S1.doc]

|  | **Customised orthoses** | **Prefabricated Orthoses** |
| --- | --- | --- |
| **Pre-manufacture preparation** |  |  |
| Casting | Yes | No |
| Measurements for  individualised prescription | Yes | No |
| **Characteristics of the device** |  |  |
| Materials | Semi-rigid, 4mm polypropylene | Semi-rigid, 4mm polypropylene |
| Length of the device | 10-15mm proximal to the metatarsal heads | 10-15mm proximal to the metatarsal heads |
| Width of the device | *Medial border* – bisection of 1^st^ metatarsal.  *Lateral border* - lateral aspect of foot. | *Medial border* – bisection of 1^st^ metatarsal.  *Lateral border* - lateral aspect of foot. |
| Heel cup height | Approximately 12.0 mm | Approximately 12.0 mm |
| Heel post | 450kg/m^3^ ethyl vinyl acetate | 450kg/m^3^ ethyl vinyl acetate |
| Contoured arch area | Yes | Yes |
| Extrinsic heel post | Yes | Yes |
